# Childhood Obesity and Plasma Micronutrient Deficit of Chilean Children between 4 and 14 Years Old

**DOI:** 10.3390/nu15071707

**Published:** 2023-03-31

**Authors:** Oscar Castillo-Valenzuela, Lissette Duarte, Miguel Arredondo, Germán Iñiguez, Luis Villarroel, Francisco Pérez-Bravo

**Affiliations:** 1 Escuela de Nutrición y Dietética, Facultad de Medicina, Universidad Finis Terrae, Santiago 7501015, Chile; 2 Laboratorio de Nutrigenómica, Departamento de Nutrición, Facultad de Medicina, Universidad de Chile, Santiago 8331051, Chile; 3 Instituto de Nutrición y Tecnología de los Alimentos (INTA), Universidad de Chile, El Líbano 5524, Santiago 8331051, Chile; 4 Escuela de Medicina, Instituto de Investigación Materno Infantil (IDIMI), Universidad de Chile, Santiago 8331051, Chile; 5 Departamento de Salud Pública, Facultad de Medicina, Pontificia Universidad Católica de Chile, Santiago 8331150, Chile

**Keywords:** micronutrient deficiency, vitamin D status, childhood obesity

## Abstract

Objective: To analyze the nutritional status and plasma levels of vitamins and minerals in a cohort of Chilean children between 4 and 14 years old from three cities in Chile (Santiago, Antofagasta, and Concepcion). Design: This is a descriptive analysis of micronutrient levels in Chilean children as it relates to obesity and food consumption. Setting: This study included 1235 children from schools in Santiago (central area), Antofagasta (northern area), and Concepcion (southern area) in Chile. Results: Plasma levels of micronutrients revealed deficiencies in children from all these cities. Copper (26.4%) and calcium (33.0%) deficiencies were found in the children from Antofagasta, whereas iron (26.7%) and zinc (20.8%) deficiencies were found in the children from Concepcion and Santiago, respectively. The percentage of children with vitamin D deficiencies was exceptionally high in all cities (over 78%). The analysis of micronutrients and nutritional status revealed that vitamin D deficiencies were significantly higher (*p* = 0.02) in overweight children, particularly in Antofagasta. In the analysis of the nutritional status of children and their food consumption habits, the proportion of overweight and obesity was significantly higher (*p* = 0.001) in children that skipped breakfast compared to children that did not. Finally, children from low socioeconomic levels were significantly more overweight and obese compared to children from high socioeconomic levels (*p* < 0.05). Conclusions: this is the first study to describe plasma levels of micronutrients in Chilean children and adolescents. High percentages of obesity, overweight, and vitamin D deficiency were detected in children. These results are of significant relevance to future public health policies in Chile.

## 1. Introduction

In Chile, Rozowski et al. [1] reported that little information is available about the plasma levels of vitamins and minerals in Chilean children aged between 4 and 14 years. Moreover, plasma micronutrient levels have not been documented in the Chilean population since 1960 [2]. Information currently available regarding micronutrient intake was obtained using 24 h recall (24R) and food-frequency (FF) dietary surveys. Using these methods, the Chilean Food Consumption Survey (ENCA) in 2010 showed that the food intake of Chilean children was characterized by high-energy foods, low consumption of fruits and vegetables, and high consumption of bread [3]. Thus, according to the guidelines used by the ENCA, 95% of the Chilean population has a poor diet.

Childhood obesity is one of Chile’s primary public health problems [4]. The latest report of the Food and Agriculture Organization for the United Nations (FAO) indicated that Chile had one of the highest prevalences of overweight in Latin America and the Caribbean, with a prevalence of 9.3% of overweight children under five years of age [5]. Additionally, according to data published by the Chilean Aid and Scholarship Board (JUNAEB) in 2020, 25% of children and adolescents were obese and 29% were overweight [6]. Similarly, the latest data published by the Organization for Economic Cooperation and Development (OECD) showed that 74% of Chilean adults over 15 were overweight or obese. This is the highest prevalence in the region, ahead of Mexico (72.5%) and the United States (71%) [7]. These percentages are relevant, as overweight is one of the determining factors responsible for chronic non-communicable diseases such as diabetes, cardiovascular disease, and hypertension, representing a high economic cost for these countries [8].

There are few studies in Chile in the last 30 years that have included biochemical markers of nutritional status and food intake. This lack of information hinders the definition of strategies and policies at the governmental level to address malnutrition (for deficit or excess). The most recent data are limited to vitamin D only and show a deficiency, mainly in the southern areas of Chile. It is then necessary to corroborate results obtained from 24 h dietary recall surveys with biochemical measurements to determine the adequacy of nutritional intake [1]. Therefore, this study aimed to analyze the nutritional status and plasma levels of vitamins and minerals in a cohort of Chilean children aged between 4 and 14 years from three cities in Chile (Santiago, Antofagasta, and Concepcion).

## 2. Methods

### 2.1. Study Design and Population

This study included 1235 children from schools in Santiago (central area), Antofagasta (northern area), and Concepcion (southern area) in Chile. The sample size was calculated based on data published about deficiencies of vitamin D in Chile and the size of the targeted population based on the estimation of the Chilean Statistics Institute (INE) in 2016 [9]. Using a standard error of 5% and a confidence interval of 95%, a total of 419 children from Santiago, 406 children from Concepcion, and 410 children from Antofagasta were included in our study. Children were randomly selected by age group and socioeconomic level from public and private schools. The ethics committee of the Institute of Nutrition and Food Technology of the University of Chile approved the study—approval Code: P04-2018; approval date: 24 January 2018. Parents and guardians signed informed consent forms and schoolchildren gave their consent for nutritional and biochemical evaluations. Children diagnosed with chronic diseases that could interfere with food consumption were excluded from this study.

### 2.2. Survey Design and Application

A modified food-frequency survey was applied to examine the frequency of food consumption by children in this study, including healthy and unhealthy foods that are most frequently consumed by children (the survey is available as Supplementary Materials). This survey was previously validated in schoolchildren in Chile [10]. The survey was administered directly to children 8 years of age and older, whereas for children under eight years of age, the survey was administered to their parents. Through this survey, the daily consumption of fruits, vegetables, dairy products, and sugary drinks, in addition to the weekly consumption of fish, vegetables, hot dogs, hamburgers, pizzas, candies, and sweet and salty snacks was evaluated. Snack items were also included and children were asked about the money they bring to school and the type of snack that was sent from home. In addition, questions related to screen and leisure time were included in the survey.

### 2.3. Anthropometric Measures and Sociodemographic Characteristics

Anthropometric measurements were taken with the children in light clothing. Weight was measured on an electronic scale (SECA 813, Hamburg, Germany), and height was measured on a portable stadiometer (SECA 213, Hamburg, Germany) according to international standards. Nutritional assessment was performed according to the World Health Organization (WHO) standards for the age and gender of children [11]. Body mass index (BMI) was calculated as weight (kg)/height (m^2^). According to WHO standards, children with a BMI/age < +1 SD were classified as normal weight, those with a BMI/age between +1 standard deviation (SD) and +2 SD were classified as overweight, and those with a BMI/age > +2 SD were classified as obese. The socioeconomic level of children was determined through the ESOMAR survey. This instrument was developed in Europe [12] and it has been previously used in Chile [13]. This survey uses the educational level and occupation of the head of the household to perform a stratification of the socioeconomic level. The children who participated in this study come from public and private schools. The schools were randomly selected and represented the country’s educational structure.

### 2.4. Blood Samples

A single 4 mL blood sample was taken in tubes with EDTA. Fasting was not requested prior to sample collection. The samples were taken in the morning (09:00 to 12:00 h). The samples were then immediately centrifuged at 1200× *g* for 15 min. Plasma was stored in aliquots of 400 µL in cryotubes that were stored at −20 °C until the micronutrient determinations were made. The sample collection was conducted from May to August 2018 in the three cities.

### 2.5. Biochemistry Analyses

#### 2.5.1. Plasma Metal Measurement

A flame atomic absorption spectrum (Perkin Elmer 2280) was used for zinc determination. All samples were diluted 1 in 5. A zinc standard solution of 1000 mg/L was used as the standard (Cat Nº: 1.19806.0500, Merck, Darmstadt, Germany). The determinations of iron and copper were performed in an atomic absorption spectrum with a Graphite Furnace (Perkin Elmer Simaa 6100). The samples were diluted 1 in 100. A five-point calibration curve for each metal was performed using a CertiPur solution for Fe (1.19781), Cu (1.9786), and Zn (1.9806) (Merck, Darmstadt, Germany). As an internal control, MR-CCHEN-002 (Venus Antiqua) and Dolt-2 (Dogfish liver) preparations were used as reference materials to validate the mineral analyses. Calcium determination was carried out using the Calcio Arsenazo III kit (Química Clínica Aplicada S.A., Amposta, Spain) according to the kit instructions. It was read in a UV-Vis Spectrophotometer (UV-1700, Pharmaq Spec, Shimadzu Corp., Kyoto, Japan) at 650 nm. A cutoff of 70 µg/dL as the lower-normal limit for serum iron, zinc, and copper, and 8.0 mg/dL for calcium were used. All materials used to take blood samples, process plasma separation, and process samples were metal-free.

#### 2.5.2. Vitamin Measurement

Measurements of vitamin B12 were performed using a commercial kit (VITROS 3600; San Diego, CA, USA), which included a pretreatment of the sample that allows vitamin B12 to be released from its binding to endogenous proteins to perform an enzyme-linked immunosorbent assay (sensitivity: 10 pg/mL, intra-assay CV: 2.0%, inter-assay CV: 4.8%). Vitamin A and E were measured by HPLC using a commercial kit (ChromSystems; Munich, Germany) and by UV detection at 295 nm using a solvent degasser to have a stable baseline (sensitivity: 0.02 mg/L and 0.60 mg/L, respectively). The intra-assay CV was 1.0% and the inter-assay CV was 4.5% for vitamin A, and 2.1% and 3.8% for vitamin E, respectively. Vitamin D was measured using a commercial ELISA Kit (DiaSource, Louvain, Belgium) with a sensitivity of 2 ng/mL, an intra-assay CV of 5.1%, and an inter-assay CV of 5.3%.

### 2.6. Statistical Analysis

Data are presented as means, standard deviations, and percentages. The normality assumption was tested using the Shapiro–Wilk test. A one-way analysis of variance (ANOVA) was used for the normally distributed variables. The Kruskall–Wallis test was used when the normality criteria were not met. The chi-square test was used to detect the association between the categorical variables. A significant result was considered at *p* < 0.05. Data were processed in an Excel spreadsheet (Microsoft Office 2016) and then analyzed with SPSS 19.0 (IBM Inc., New York, NY, USA).

## 3. Results

### 3.1. Physical Characteristics of the Study Group

**Figure 1:**
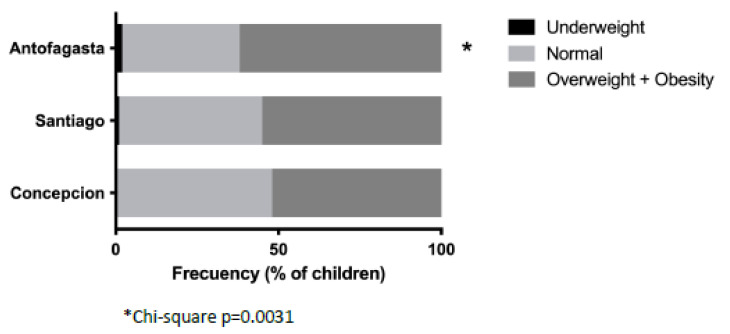
Distribution of nutritional status among cities. * Higher prevalence of overweight and lower prevalence of normal nutritional status in children between Antofagasta and the other two cities.

On average, the weight, height, and BMI of children from Antofagasta were significantly higher than children from Santiago and Concepcion. On the other hand, children from Santiago were smaller and had their weight and height were lower. (Table 1). Children from Antofagasta also had a significantly lower prevalence of “normal” nutritional status compared to children from Santiago and Concepcion (Figure 1). In each city, over half of the children were overweight and obese. In Antofagasta, the prevalence of overweight and obese children was 61.9%, which was significantly higher than in Santiago and Concepcion (Figure 1).

### 3.2. Analysis of Micronutrients

**Figure 2:**
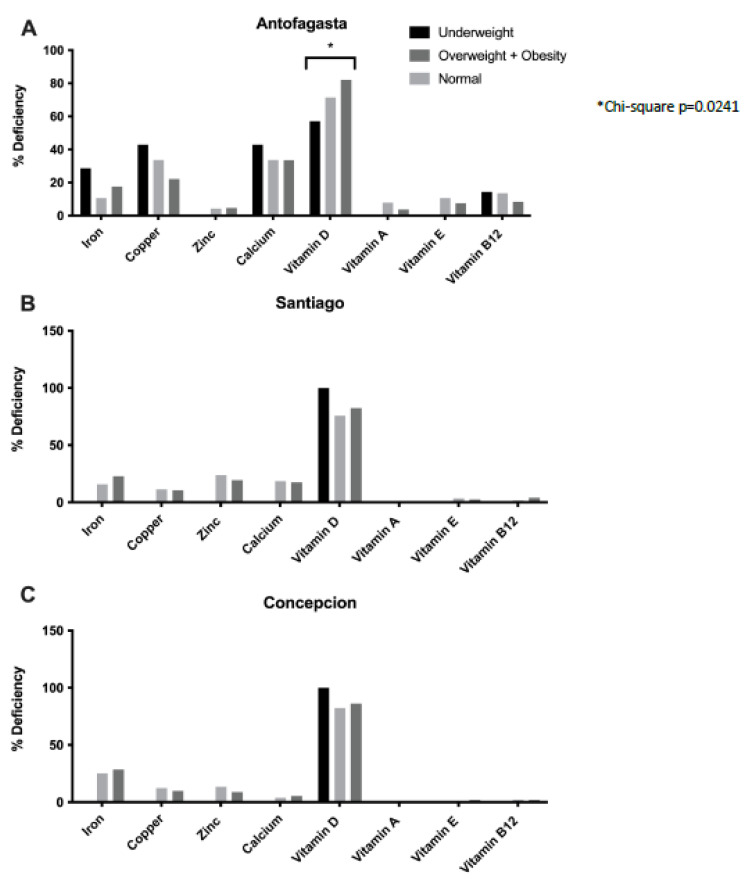
Plasma micronutrient deficiency according to nutritional status in (**A**) Antofagasta, (**B**) Santiago, and (**C**) Concepcion. (* significant difference in nutritional status).

The percentage of children from each city with deficiencies in plasma levels of micronutrients is presented in Table 2. Children from all cities revealed deficiencies in micronutrients. In Antofagasta, children had significantly higher deficiencies in copper (26.4%) and calcium (33.0%). Iron deficiency was significantly higher in children from Concepcion (26.7%), whereas zinc deficiency was significantly higher in children from Santiago (20.8%). The percentage of children with deficiencies in vitamin D was exceptionally high in all cities. This deficiency was higher in Concepcion than in Antofagasta and Concepcion (borderline significance). For all other vitamins, the percentage of children with deficiencies was not different across the cities, except for Antofagasta, where children showed a significantly higher percentage of deficiencies in vitamins B12, E, and A compared to children from Santiago and Concepcion. The analysis of the micronutrients and the nutritional status revealed that deficiencies in vitamin D were significantly higher (*p* = 0.02) in overweight children, particularly those from Antofagasta (Figure 2). No other differences in vitamin deficiencies were detected.

Children from Antofagasta showed the most significant percentages of vitamin deficiencies compared to children from Santiago and Concepcion. In the analysis of microminerals, the comparison by sex revealed no differences. Children of both sexes from Antofagasta had the highest deficiencies in calcium compared to children from Santiago and Concepcion.

### 3.3. Food Consumption and Lifestyle Habits

The survey results revealed that 75% of children consume sugary drinks daily, 61.1% consume sweets daily, 70% do not consume at least one fruit daily, 65% do not consume dairy products daily, and 50% do not consume vegetables daily. In addition, the study found that 40% of children and adolescents had more than two hours of screen time each day (Supplementary Materials).

**Figure 3:**
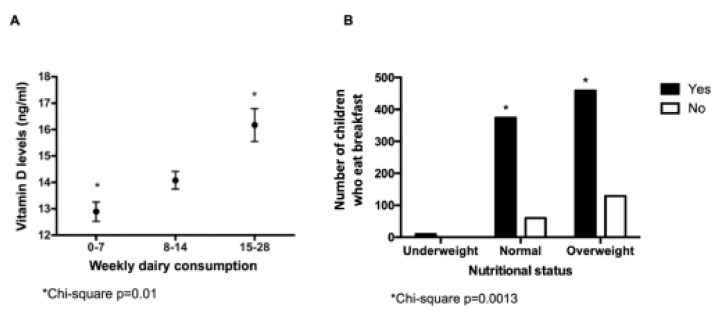
(**A**) Vitamin D levels according to average weekly dairy consumption. (**B**) Eating breakfast according to the nutritional status of children and adolescents included in the study.

Regarding food consumption habits and plasma levels of vitamins and minerals (Figure 3), the higher the consumption of dairy products, the higher the plasma levels of vitamin D (*p* = 0.01). No other significant association was detected for other foods or groups of food.

In the analysis of children’s nutritional status and food consumption habits, the proportion of overweight and obesity was significantly higher (*p* = 0.001) in children that skipped breakfast compared to children that did not (Figure 3B). No other significant associations between food consumption habits and nutritional status were detected.

A significant association between the socioeconomic level of children and nutritional status was detected. Children from low socioeconomic levels were significantly overweight and obese compared to children from high socioeconomic levels (*p* < 0.05). An inverse relationship between plasma levels of vitamin D and screen time was detected, where the higher the amount of screen time, the lower the plasma levels of vitamin D (*p* < 0.05).

## 4. Discussion

Over the last few decades, there has been an explosive increase in the population of overweight and obese children in Chile [6]; however, no information was available regarding the nutritional status and concentrations of plasma levels of micronutrients in this population. Therefore, our study aimed to examine the nutritional status and plasma levels of vitamins and minerals in children from three Chilean cities. Under FAO’s vision, our study selected the vitamins and minerals that are traditionally a nutritional problem in Latin America, that is, vitamins A and D and the micronutrients calcium, iron, and zinc.

The results of the nutritional status of the group of children included in our study were similar to those of previous studies on the Chilean population. The prevalence of low height in our study group was 1.3%. The results of our study revealed that the prevalence of overweight and obese children was higher in Antofagasta. Although the high prevalence of overweight and obese children in both regions of Chile (Antofagasta and Concepcion) has already been reported, the exceptionally high prevalence detected in Antofagasta was unexpected [6]. There are different results when comparing the nutritional status of children from Chile and other Latin-American countries. For example, the group of countries with a high prevalence of “low height”, overweight, and obesity includes Bolivia, Ecuador, Honduras, and Nicaragua. Other countries that are similar to Chile, with a moderate prevalence of “low height” children and a high prevalence of overweight and obesity, include Brazil and Mexico [14].

From the results of our study, it is interesting to note that the high prevalence of overweight and obese children was associated with deficiencies in the plasma levels of micronutrients. This phenomenon is known as the double burden of malnutrition in which individuals are overweight or obese but simultaneously have nutritional deficits. Our findings are consistent with those of Iglesias et al. (2019) and Shimabuku et al. (2020), which showed a high proportion of overweight and obese individuals and a high prevalence of individuals with anemia due to iron deficits in several Latin-American countries [15,16].

In our study group, we detected high percentages of children with deficiencies in plasma levels of vitamin D and iron across all cities, as well as copper and zinc deficiencies in Antofagasta and Santiago, respectively. The results of the National Health Survey in Chile in 2016–2017 [17] also revealed that 15% of fertile women and 20% of older adults had levels of 25 (OH) vitamin D lower than 20 ng/mL. Another recent study in Chilean older adults reported that 88% of older adults had deficiencies in 25 (OH) vitamin D [18]. However, a review study published in Chile in 2015 [1] concluded that little information was available regarding the levels of micronutrients in the Chilean pediatric population. Only two studies have been published about individuals from two cities (Coyhaique and Punta Arenas) in southern Chile (latitude 45.34° and 53.93°, respectively). These studies reported a high prevalence of overweight and obesity in both cities, where 61% and 96% of children in Coyhaique and Punta Arenas had deficiencies in 25 (OH) vitamin D, respectively [19,20]. In other Latin-American countries closer to Ecuador than Chile, the prevalence of children with 25 (OH) vitamin D deficiencies was lower. For instance, this prevalence was 12% in Colombia, 24% in Mexican preschoolers, and 31% in Brazilian children aged between 11 and 15 months [21,22,23].

Regarding deficiencies in iron, the Chilean population has higher plasma levels of iron compared to the populations of other Latin-American countries. Anemia caused by iron deficiencies in Chilean preschool- and school-aged children is almost nonexistent [24]. Several policies have contributed to this reduction, including the improvement of sanitary conditions and nutritional education, as well as the fortification initiatives of the Complementary National Feeding Program (PNAC) [25]. There is recent evidence about the prevalence of iron deficiency and anemia in Chile. For example, preliminary data in preschoolers from the south of Santiago (La Pintana, Chile) in 2001 showed a prevalence of anemia of 1%. At the beginning of the 1990s, the prevalence of anemia ranged from 0% to 1.2% in Chile [26].

A study conducted in the mid-1990s revealed that administering physiologic doses of zinc had a positive effect on height in male preschoolers from the northern area of Santiago, Chile [27]. Another study in the Chilean population confirmed low and moderate zinc deficiencies in children and adolescents [28]. However, it has been suggested that the impact of zinc deficiencies on the height of children and adolescents is almost nonexistent. It may be that the results reported in this study revealed a different reality in which lower socioeconomic levels and the influence of recent immigration to Chile (Haiti, Peru, Bolivia, and Venezuela) are significant factors that have not yet been studied.

Copper deficiencies have been poorly investigated in Chilean school-aged children. There are no studies available regarding measures of plasma levels of copper. It is important to note that reduced levels of copper in serum are not typically reported because the normal body mechanisms of copper regulation are extremely sensitive and can protect against deficiencies and excesses [29].

Latin-American studies published many years ago showed that deficiencies in vitamin B12 in children aged from 8 to 12 years ranged from 11% to 22% for marginal levels of B12 in Guatemala [30]. In Venezuela, the incidence of vitamin B12 deficiency was 11.4% and the most significant deficiencies were found in preschool-aged (35%) and school-aged (27.5%) children [31]. In Chile, information about vitamin B12 is scarce and data from 1985 revealed a deficiency in folates (1.2%) in school-aged children [32].

In Chile, there are no studies available about the plasma levels of vitamins A and E in school-aged children For vitamin A, our results showed that in school-aged children, the level of vitamin A was adequate or as expected; therefore, the dietary requirements are being met. For vitamin E, the situation was similar and the deficiency appeared to be localized in the northern area of Chile (Antofagasta). The immigration rate in this region (from Perú, Haiti, and Venezuela) could be relevant. The evidence from other Latin-American countries indicates that Chile has a very low prevalence of vitamin A deficiency [33].

The results of our study on the nutritional status and deficiencies in the plasma levels of vitamins and minerals in Chilean children align with the feeding pattern of the age group studied. This feeding pattern is characterized by high consumption of sugary drinks and sweets and low consumption of fruits, vegetables, and dairy products. However, we must consider that this information was obtained through an FFQ, which has some limitations such as the use of memory and tends to overestimate intake.

This diet is similar to that found in previously reported studies on Chilean children [1,13,14], and it may indicate that food consumption habits have not changed. It is challenging to change these food consumption habits and it seems necessary to tackle this challenge from different perspectives. Regarding the association between overweight, obesity, and the habit of skipping breakfast, a systematic review of Antofagasta reported similar results, mainly in adolescents [34], revealing higher frequencies of several deficiencies. In the last seven years, this region has been exposed to an influx of immigrants, likely with different food consumption habits and cultures. However, unfortunately, there are no studies that demonstrate the impact of this variable on the epidemiology pattern of obesity.

From the point of view of water quality, studies from 2006 to 2016 showed that the drinking water quality in Antofagasta was acceptable based on the guidelines because there were no significant differences observed between the types of water and only a few minor breaches were observed compared with other regions in Chile. In our study, the population was derived from urban zones where the drinking water quality is similar to that of other countries [35].

Finally, there was little evidence in our study to demonstrate the association between screen time and vitamin D levels, although Chilean children are less exposed to daylight when spending more time on screens. A multicentric study in Brazilian children revealed that higher levels of physical activity were associated with higher plasma concentrations of vitamin D. However, they did not report any associations with excessive screen time [36].

## 5. Conclusions

This is the first study to describe the plasma levels of micronutrients in Chilean children and adolescents. We observed a high prevalence of obesity, overweight, and poor eating habits. In addition, deficiencies in vitamin D and, to a lesser extent, copper, calcium, and zinc, were detected. Vitamin D deficiency was found to be the highest in overweight children. This study demonstrates that the determination of micronutrient levels in different populations every few years should be a standard practice in research, where the objective, beyond describing a specific situation, is to impact decision making (Ministry of Health), resulting in revisions of public policies.

## Figures and Tables

**Table 1:** Physical characteristics of children by city.

	Antofagasta (n = 410)	Santiago (n = 419)	Concepcion (n = 406)	*p*-Value
Age (years)	10.1 ± 2.7 _(a)_	9.5 ± 2.7 _(b)_	10.0 ± 2.8 _(a,b)_	0.010
Weight (Kg)	46. ± 17.0 _(a)_	40.1 ± 15.7 _(b)_	42.9 ± 15.9 _(b)_	0.001
Height (cm)	143.0 ± 16.7 _(a)_	139.36 ± 17.5 _(b)_	142.6 ± 17.2 _(c)_	0.012
Waist circumference (cm)	69.9 ± 12.0 _(a)_	64.2 ± 10.6 _(b)_	68.2 ± 11.4 _(a)_	0.000
BMI (Kg/m^2^)	21.7 ± 4.7 _(a)_	19.8 ± 3.8 _(b)_	20.3 ± 4.1 _(b)_	0.000
zBMI/age	1.2 ± 1.1	1.1 ± 1.1	1.05 ± 1.1	0.320
zHeight/age	0.50 ± 1.1 _(a)_	0.29 ± 1.1 _(b)_	0.11 ± 0.9 _(c)_	0.000

BMI: body mass index. a, b, and c, indicate significant differences between cities (ANOVA and Tukey post hoc test).

**Table 2:** Percentage of Chilean children with plasma level deficits of micronutrients by city.

	Region			
Micronutrients	Antofagasta (n = 410)	Santiago (n = 419)	Concepcion (n = 406)	*p*-Value
Iron (<70 µg/dL)	15.1 _(a)_	19.8 _(a)_	26.7 _(b)_	<0.001 *
Copper (<70 µg/dL)	26.4 _(a)_	10.8 _(b)_	11.6 _(b)_	<0.001 *
Zinc (<70 µg/dL)	4.4 _(a)_	20.8 _(b)_	11.3 _(a)_	<0.001 *
Calcium (<8.8 mg/dL)	33.0 _(a)_	17.7 _(b)_	4.7 _(b)_	<0.001 *
Vitamin D (<29.9 ng/mL)	78.5	78.9	84.4	0.058
Vitamin A (<12 years of age: <0.20 µg/L; >12 years of age: <0.26 µg/L)	4.9 _(a)_	0 _(b)_	0.5 _(b)_	<0.001 *
Vitamin E (<12 years of age: <4.0 mg/L; >12 years of age: <6.0 mg/L)	8.2 _(a)_	3.2 _(b)_	1.5 _(b)_	<0.001 *
Vitamin B_12_ < 187 pg/mL	9.8 _(a)_	2.9 _(b)_	2.0 _(b)_	<0.001 *

Chi-square (* *p* < 0.001). a, b, indicate significant differences between cities.

## Data Availability

The data of the present study are available for further scientific analysis from the corresponding author on reasonable request.

## Supplementary Materials

The following supporting information can be downloaded at: https://www.mdpi.com/article/10.3390/nu15071707/s1, File S1: School survey.
