# Supplementary material for: Childhood Obesity and Plasma Micronutrient Deficit of Chilean Children between 4 and 14 Years Old
Source: Nutrients. 2023 Mar 31;15(7):1707. doi: 10.3390/nu15071707 (PMC10096594; doi:10.3390/nu15071707)
Supplement: Supplementary file 1 [file nutrients-15-01707-s001.zip › nutrients-2291092-supplementary.pdf]

School Survey

Name____________________________________ Date __/__/__ Sex/Gender ____

Grade ___-___ School: ___________________________________

Please, answer the following questions:

1. How many days a week do you consume milk, yogurt or cheese?

☐ None ☐ 1 ☐ 2 ☐ 3 ☐ 4 ☐ 5 ☐ 6 ☐ 7

2. How much dairy, such as milk, cheese, yogurt, do you eat per day?

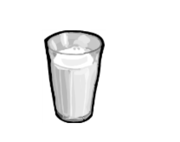	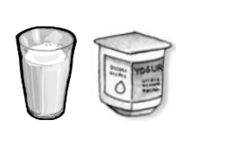	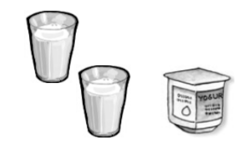	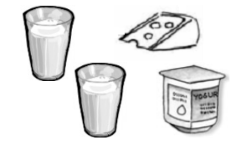		I don't eat dairy products
1 portion	2 portions	3 portions	4 portions	nº de portions	

3. How many days a week do you eat fruit like apple, banana, pear, orange...?

☐ None ☐ 1 ☐ 2 ☐ 3 ☐ 4 ☐ 5 ☐ 6 ☐ 7

4. How many fruits do you eat a day?

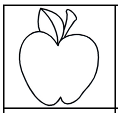	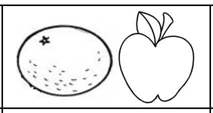	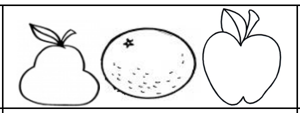	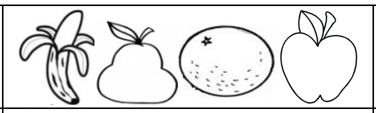		I don't eat fruits
1 fruit	2 fruits	3 fruits	4 fruits	n° de fruits	

5. How many days a week do you eat vegetables or salads?

☐ None ☐ 1 ☐ 2 ☐ 3 ☐ 4 ☐ 5 ☐ 6 ☐ 7

6. How many vegetable dishes or salads do you eat a day?

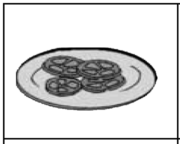	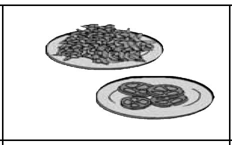	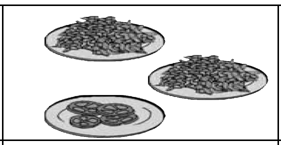	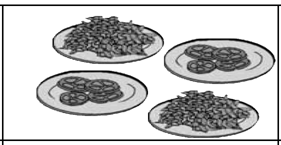		I don't eat salads or vegetables
1 plate	2 plates	3 plates	4 plates	nº of plates	

7. How many glasses a day do you drink of juices and/or soft drinks, normal or with sugar (not light or zero)?

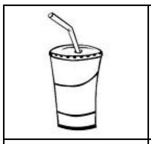	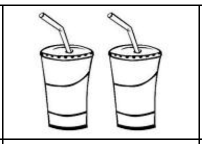	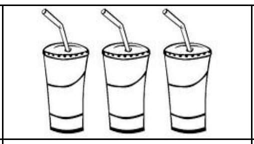	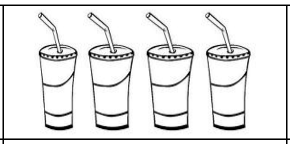		I don't drink juices and fizzy drinks
1 glass	2 glasses	3 glasses	4 glasses	n° glasses	

8. How many glasses of water do you drink a day?

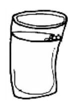	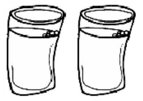	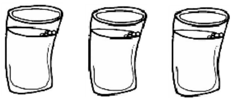	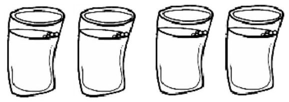		I don't drink water
1 glass	2 glasses	3 glasses	4 glasses	n° glasses	

9. How many times a week do you eat legumes such as lentils, beans or chickpeas?

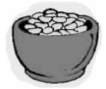	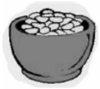	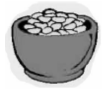	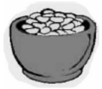		I don't eat legumes
1 time per week	2 times per week	3 times per week	4 times per week	n° times per week	

10. How many times a week do you eat fish?

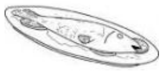	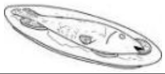	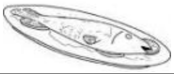	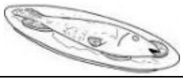		I do not eat fish
1 time per week	2 times per week	3 times per week	4 times per week	n° times per week	

11. How many times a week do you eat cookies or candies?

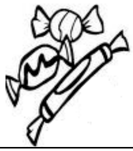	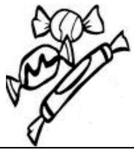	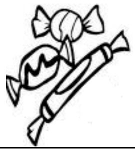	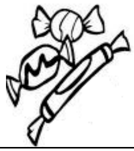		I don't eat cookies or candies
1 day a week	2 days a week	3 days a week	4 days a week	n° days per week	

12. How many times a week do you eat French fries, pizzas or burgers?

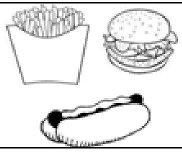	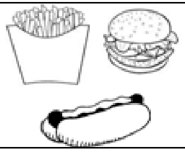	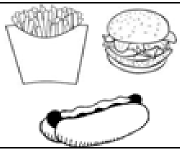	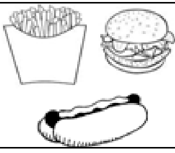		I don't eat fries, pizzas, burgers
1 day a week	2 days a week	3 days a week	4 days a week	n° days per week	

13. How many times a week do you eat salty products such as chips or nachos?

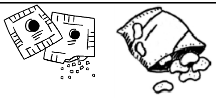	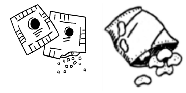	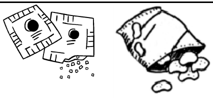	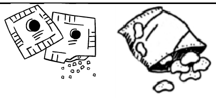		I don't eat salty snacks
1 day a week	2 days a week	3 days a week	4 days a week	n° days per week	

1. Do you eat a snack at school? ☐ YES ☐ NO
2. Do you bring a snack from home? ☐ YES ☐ NO
3. Do you buy snacks at school or outside of school? ☐ YES ☐ NO
4. Do they give you special snacks at school (cookie, cereal, cake, etc.)? ☐ YES ☐ NO

5. What do you bring from home for lunch? (Ask if he answered yes to question 15)

- ☐ Fruits
- ☐ Vegetables
- ☐ Salty snacks, chips, soufflés, etc.
- ☐ Milk or yogurt
- ☐ Sweet snacks, cookies, chocolates, etc.
- ☐ French fries, empanadas, etc
- ☐ Bread, crackers, etc
- ☐ Sugary drinks or juices
- ☐ Sugar-free drinks or juices
- ☐ Hard-boiled egg
- ☐ Water

6. Do you bring money to buy food at school?

- ☐ 1 day a week
- ☐ 2 days a week
- ☐ 3 days a week
- ☐ 4 days a week
- ☐ 5 days a week

7. How much money do you bring to school? $ ____________

8. What do you buy with the money?

- ☐ Fruits
- ☐ Vegetables
- ☐ Salty snacks, chips, soufflés, etc.
- ☐ Milk or yogurt
- ☐ Sweet snacks, cookies, chocolates, etc.
- ☐ French fries, empanadas, etc
- ☐ Bread, crackers, etc
- ☐ Sugary drinks or juices
- ☐ Sugar-free drinks or juices
- ☐ Hard-boiled egg
- ☐ Water

9. What do you usually do at recess?

- ☐ You play ball, run, jump rope or similar
- ☐ You sit down (to talk, read, play mobile games or similar)
- ☐ You sit down to eat

10. What do you usually do after school?

- ☐ Workshop or sports activity such as soccer, basketball, karate, athletics, zumba, others
- ☐ Play in the square or street
- ☐ Watch TV or play video games
- ☐ Riding a bike, rollerblading

11. Outside of school, how many hours a day do you watch television or play on the computer/console/cell phone?

- ☐ Less than 1 hour
- ☐ 1 to 2 hours
- ☐ 2 to 3 hours
- ☐ More than 3 hours
